# Role of Surface Area, Primary Particle Size, and Crystal Phase on Titanium Dioxide Nanoparticle Dispersion Properties

**DOI:** 10.1007/s11671-010-9772-1

**Published:** 2010-09-03

**Authors:** Komkrit Suttiponparnit, Jingkun Jiang, Manoranjan Sahu, Sirikalaya Suvachittanont, Tawatchai Charinpanitkul, Pratim Biswas

**Affiliations:** 1 Aerosol and Air Quality Research Laboratory, Department of Energy, Environmental & Chemical Engineering, Washington University in St. Louis, St. Louis, MO 63130, USA; 2 Department of Chemical Engineering, Kasetsart University, 50 Paholyothin Road, Jatujak, Bangkok 10900, Thailand; 3 Center of Excellence in Particle Technology, Department of Chemical Engineering, Faculty of Engineering, Chulalongkorn University, Patumwan, Bangkok 10330, Thailand

**Keywords:** Nanoparticle dispersion, Titania, Ionic strength, Isoelectric point, Nanotoxicology

## Abstract

Characterizing nanoparticle dispersions and understanding the effect of parameters that alter dispersion properties are important for both environmental applications and toxicity investigations. The role of particle surface area, primary particle size, and crystal phase on TiO_2_ nanoparticle dispersion properties is reported. Hydrodynamic size, zeta potential, and isoelectric point (IEP) of ten laboratory synthesized TiO_2_ samples, and one commercial Degussa TiO_2_ sample (P25) dispersed in different solutions were characterized. Solution ionic strength and pH affect titania dispersion properties. The effect of monovalent (NaCl) and divalent (MgCl_2_) inert electrolytes on dispersion properties was quantified through their contribution to ionic strength. Increasing titania particle surface area resulted in a decrease in solution pH. At fixed pH, increasing the particle surface area enhanced the collision frequency between particles and led to a higher degree of agglomeration. In addition to the synthesis method, TiO_2_ isoelectric point was found to be dependent on particle size. As anatase TiO_2_ primary particle size increased from 6 nm to 104 nm, its IEP decreased from 6.0 to 3.8 that also results in changes in dispersion zeta potential and hydrodynamic size. In contrast to particle size, TiO_2_ nanoparticle IEP was found to be insensitive to particle crystal structure.

## Introduction

Nanotechnology is finding applicability in the field of environmental protection and has great potential in improving air, water, and soil quality [1]. For example, engineered nanoparticles can efficiently reduce toxic metal emissions from combustion systems and improve air quality by suppressing metal vapor nucleation and promoting metal nanoparticle condensation and coagulation [2,3]. Many nanomaterials, such as TiO_2_, carbon nanotubes, and dendrimers, have been designed to degrade or absorb pollutants in water and soil systems [4-7]. These applications are often determined by the properties of nanomaterials, such as size, surface properties, crystal structures, and morphologies [8,9]. Although nanotechnology has the potential to improve the quality of the environment, there are also concerns that it can generate a new class of hazards upon release to the environment followed by exposure of either the ecosystem or human beings that may result in potential adverse effects [1,10,11]. Toxicological studies with certain engineered nanoparticles (e.g., fullerenes, quantum dots, and metal oxides) have confirmed that they can be potentially harmful due to their high surface molecule/atom fraction and unique physicochemical properties [12,13]. The emerging discipline of nanotoxicology is aiming to establish the relationship between nanoparticle properties (e.g., size, surface properties, and crystal phase) and their toxic potential [14-16].

Titanium dioxide has been widely used in environmental photocatalysis, sunscreen, and coating industry [17-19]. However, a variety of detrimental pulmonary effects in rodents and antibacterial effects have also been associated with nanosized TiO_2_ particle exposure [20-22]. Both the functionalities and biological effects of titania nanoparticles are controlled by its physicochemical properties. Nanomaterials that are tested are often dispersed in aqueous systems; this can potentially result in physicochemical property changes, e.g., agglomeration state and surface charge variation [15,23,24].

The agglomeration behavior and surface charge variation of nanoparticle dispersions can have a dramatic effect on both the reactivity of nanomaterials and their efficiency in contamination treatment [7,25,26]. It also affects the response of organisms upon exposure [27-30]. Therefore, accurate characterization of nanoparticle dispersions becomes very important for its environmental applications and nanotoxicology investigations. Jiang et al. [15] characterized the state (such as the hydrodynamic size, surface charge, and the degree of agglomeration) of titania and other nanoparticle suspensions and tested the effect of solution pH and ionic strength (IS) on dispersion properties. However, this study involved only a single value of surface area, primary particle size, and crystal phase for examined dispersion state. It has been reported that these properties of TiO_2_ nanoparticle can affect its photocatalytic activity [19,31] and toxicity [16,32-34]; however, little is known about their effect on the dispersion state and agglomeration behavior. There is evidence suggesting that the point of zero charge of hematite nanoparticle dispersion might change with varying particle size [35]. However, systematic investigations for titania nanoparticle dispersions have not been done.

Recent developments in aerosol route synthesis of TiO_2_-based nanomaterials allow for greater and independent control of their physicochemical properties, such as size, crystal phase, and specific surface area [4,36-38]. In this study, the influence of particle surface area, primary particle size, and crystal phase on titania nanoparticle dispersion properties is investigated. TiO_2_ samples with well-controlled properties are synthesized using flame aerosol reactors (FLAR). Six anatase TiO_2_ samples with different sizes (6–104 nm) are used to study the size effect. TiO_2_ nanoparticles of different crystal phases with the same size are used to examine the crystal phase effect. Commercially available Degussa TiO_2_ (P25) sample is also tested. The effect of monovalent and divalent electrolytes is examined using sodium chloride (NaCl) and magnesium chloride (MgCl_2_).

## Materials and Methods

Several types of titania nanoparticles were used in this study. TiO_2_ (P25) nanoparticle with a primary particle size of 27 nm, specific surface area of 57.4 m^2^/g, and the phase composition of 80% anatase and 20% rutile was purchased from Degussa Chemicals (Hanau, Germany). Anatase TiO_2_ nanoparticles of 6, 16, 26, 38, 53, and 104 nm with specific surface areas of 253.9, 102.1, 61.5, 41.2, 29.7, and 15.0 m^2^/g, respectively, were synthesized using a flame aerosol reactor [16,36,39]. TiO_2_ nanoparticles of 38 nm with different crystal structures (100% anatase, 49% anatase/51% rutile, and 36% anatase/63% rutile) and a specific surface area of 41.2 m^2^/g were also synthesized in the flame aerosol reactor. The properties of these samples have been characterized using different techniques, including X-ray diffraction, transmission electron microscopy, and BET adsorption. They are reported in our previous studies [14,34] and are not repeated here. The precursor used to synthesize TiO_2_ particles was titanium tetra-isopropoxide (Sigma–Aldrich, St. Louis, Missouri). Rutile TiO_2_ particle with the primary particle size of 102 nm and a specific surface area of 13.8 m^2^/g was prepared by annealing flame-synthesized anatase TiO_2_ at size 53 nm in a furnace [16]. Other chemicals used in this study including sodium chloride (NaCl), magnesium chloride (MgCl_2_), sodium hydroxide (NaOH), and hydrogen chloride (HCl) were obtained from Sigma–Aldrich (St. Louis, Missouri).

The hydrodynamic size and surface charge (zeta potential) of nanoparticle dispersions were characterized using the ZetaSizer Nano ZS (Malvern Instruments Inc., UK), utilizing dynamic light scattering (DLS) and electrophoretic light scattering (ELS), respectively [40]. DLS measures the intensity of the laser light that is scattered from dissolved macromolecules or suspended particles. The dispersion hydrodynamic diameter is derived from the temporal evolution of the scattered light intensity using the Stokes–Einstein equation [15]. ELS measures the frequency or phase shift of an incident laser beam caused by electric field driven particle migration, reported as the electrophoretic mobility. Particle zeta potential is calculated from the measured electrophoretic mobility using the Smoluchowski equation [15,41].

The experimental plan is summarized in Table 1. To examine the effect of solution ionic strength (IS) and pH on the hydrodynamic size, surface charge, and isoelectric point (IEP), TiO_2_ (P25) was dispersed in NaCl solutions with different molar concentrations and the solution pH was adjusted by adding HCl and NaOH (case 1). To determine the effect of monovalent and divalent electrolytes on the hydrodynamic size and zeta potential, TiO_2_ (P25) was dispersed in solutions with a certain IS and molar concentration (case 2). NaCl and MgCl_2_ were employed as the monovalent and divalent electrolyte, respectively. In case 3, TiO_2_ (P25) was dispersed in deionized (DI) water with different particle concentrations to test the influence of surface area on dispersion properties. Solutions with pH of 4 and IS of 0.001–0.1 M were also used. In case 4, flame-synthesized anatase TiO_2_ of different sizes (6–104 nm) were dispersed in DI water and solutions with different pH values to study the role of primary particle size on IEP. The crystal phase effect was examined using synthesized TiO_2_ nanoparticles with different crystal structures (case 5). Typically, dilute dispersions are used in toxicological studies to represent realistic exposure scenarios. Therefore, the particle concentration tested in this study was in the range of 15–500 μg/ml. In all experiments, titania nanoparticle dispersions were sonicated for 15 min using a bath sonicator (40 W, 50 kHz, Fisher Scientific, Fairlawn, New Jersey) before the size and zeta potential measurement. All measurements were carried out at 25°C, which was maintained by the Zetasizer instrument. Repeatability of all hydrodynamic size and zeta potential was verified with more than five measurements.

**Table 1:** Summary of experiments performed

Case	Nanoparticles	Conditions	Objective
1	TiO_2_ (P25)	Particle concentration: 50 μg/ml; Three ionic strengths (0.001, 0.01, and 0.1 M) and varying pH (3–11) by adding HCl, NaCl, and NaOH.	Determine the effect of solution IS and pH on dispersion characteristics
2	TiO_2_ (P25)	Particle concentration: 50 μg/ml; NaCl and MgCl_2_ with the same IS and with the same molar concentrations.	Examine the effect of electrolyte type (monovalent vs. divalent) on dispersion characteristics
3	TiO_2_ (P25)	Particle concentration: 15, 25, 50, 150, and 500 μg/ml; DI H_2_O; Solutions with pH of 4 and IS of 0.001–0.1 M by adding HCl and NaCl.	Test the effect of nanoparticle surface area (mass concentration) on the dispersion properties
4	Anatase TiO_2_ (6–104 nm)	Particle concentration: 50 μg/ml; DI H_2_O; Solutions with IS of 0.001 M and varying pH (3–11) by adding HCl, NaCl, and NaOH.	Study the effect of primary particle size on dispersion properties
5	TiO_2_ (varying crystal phases)	Particle concentration: 50 μg/ml; Solutions with IS of 0.001 M and varying pH (3–11) by adding HCl, NaCl, and NaOH.	Investigate the effect of crystal phase on dispersion isoelectric point (IEP)

## Results and Discussion

Surface charge and hydrodynamic diameter are two important properties of nanoparticle dispersions. When a nanoparticle is dispersed in an aqueous solution, surface ionization and the adsorption of cations or anions result in the generation of the surface charge and an electric potential will be developed between the particle surface and the bulk of dispersion medium [42,43]. Depending on the measurement technique, surface charge can be represented by either the surface charge density (potentiometric titration) or the zeta potential (electrokinetic methods). The point where surface charge density equals zero is defined as point of zero charge (PZC), while the point where zeta potential equals zero is defined as isoelectric point (IEP) [41,44]. The surface of TiO_2_ nanoparticles dispersed in water is generally covered by hydroxyl group as shown in Eq. 1,

(1) $${\text{Ti}}^{\text{IV}}+{\text{H}}_{2}\text{O}\to {\text{Ti}}^{\text{IV}}-\text{OH}+{\text{H}}^{+}$$

The surface charge of titania is a function of solution pH, which is affected by the reactions that occur on the particle surface as shown in Eqs. 2 and 3,

(2) $${\text{Ti}}^{\text{IV}}-\text{OH}+{\text{H}}^{+}\to {\text{Ti}}^{\text{IV}}-{\text{OH}}_{2}^{+}$$

(3) $${\text{Ti}}^{\text{IV}}-\text{OH}\to {\text{Ti}}^{\text{IV}}-{\text{O}}^{-}+{\text{H}}^{+}$$

The pH at which the surface of titania is neutral is point of zero charge or isoelectric point. If no specific adsorption of the ions presented in the solution takes place on the particle surface, the pH at PZC and IEP would be the same. When pH is less than pH_PZC_ (pH_IEP_), Eq. 2 results in creation of the positive surface charge and positive zeta potential. When pH is larger than pH_PZC_ (pH_IEP_), Eq. 3 results in creation of the negative surface charge and negative zeta potential [42,43,45]. The dispersion hydrodynamic diameter is controlled by nanoparticle agglomeration in the aqueous system. In the classical Derjaguin–Landau–Verwey–Overbeek (DLVO) theory, the agglomeration of nanoparticles is determined by the sum of the repulsive electrostatic force (the interaction of electrical double layer surrounding each nanoparticle) and the attractive van der Walls force [46,47]. Increase in particle surface charge (zeta potential) can enhance the electrostatic repulsive force, suppress the agglomeration, and subsequently reduce dispersion hydrodynamic size.

The effects of solution pH and ionic strength (IS) and electrolyte type on titania dispersion properties are presented first, followed by discussion about the influence of particle surface area. Finally, both primary particle size and crystal phase effect on dispersion characteristics are examined.

### pH and IS Effect

**Figure 1:**
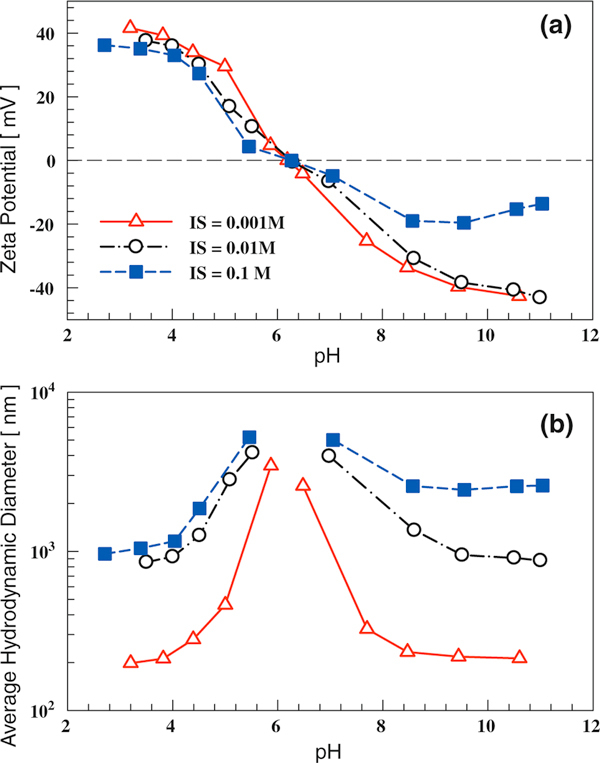
**The influence of solution ionic strength (IS) and pH on TiO_2_ (P25) dispersion properties: a zeta potential; b hydrodynamic diameter**.

The effect of solution pH and ionic strength (IS) on the zeta potential and hydrodynamic size is shown in Figure 1. The IEP for TiO_2_ (P25) is approximately 6.2, which is consistent with those reported in other studies [15,48,49]. Since NaCl is an inert electrolyte for TiO_2_ dispersion (no specific adsorption of Na^+^ or Cl^-^ by the titania nanoparticle), the IEP remains the same at different ionic strengths obtained by varying the NaCl concentration [41,50]. When pH is different from pH_IEP_, an increase in IS reduces the dispersion zeta potential by compressing the electrical double layer. This is consistent with previous tests [15,51,52] and predictions of classical colloidal theory [53]. Solution pH affects the dispersion hydrodynamic diameter by changing the particle surface charge. Near IEP, significant agglomeration takes place; large flocs were observed, as the particle surface charge is close to zero and attractive van der Waals forces are dominant. When the pH is significantly different from IEP for titania, the absolute value of zeta potential becomes higher and the hydrodynamic size becomes smaller. Solution IS changes the dispersion hydrodynamic diameter by changing both zeta potential and electrical double layer thickness. Higher solution IS leads to a smaller electrical double layer thickness, weaker electrostatic repulsive force, and subsequently larger hydrodynamic size. The smallest hydrodynamic size observed was ~200 nm, when the solution IS was 0.001 M and pH was lower than 4.0 or higher than 8.2.

**Figure 2:**
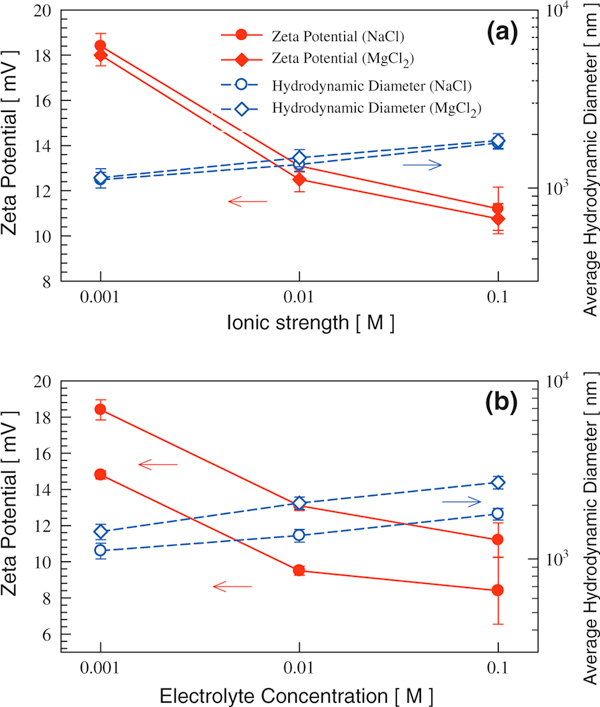
**The influence of electrolyte type (monovalent vs. divalent) on TiO_2_ (P25) dispersion properties at a the same solution ionic strength and b the same electrolyte molar concentration**.

If an electrolyte does not generate ions that can be specifically absorbed by titania nanoparticles, its influence on dispersion properties can be quantified through its contribution to solution ionic strength. TiO_2_ (P25) was dispersed in both monovalent NaCl and divalent MgCl_2_ solutions either at the same ionic strength (Figure 2a) or at the same electrolyte molar concentration (Figure 2b). The solution pH (~5.5) was lower than TiO_2_ (P25) IEP such that positive zeta potentials were observed in both cases. When the same IS was used, dispersions using NaCl and MgCl_2_ did not show any significant difference in zeta potential and hydrodynamic size. The trends of zeta potential and hydrodynamic size as a function of IS were the same as described earlier. When the same molar concentration was used, the solution IS using divalent MgCl_2_ was twice as high as the IS using monovalent NaCl. Consequently, titania dispersion using MgCl_2_ had lower zeta potential and higher hydrodynamic diameter compared to a dispersion using NaCl of the same molar concentration.

### Particle Surface Area Effect

**Figure 3:**
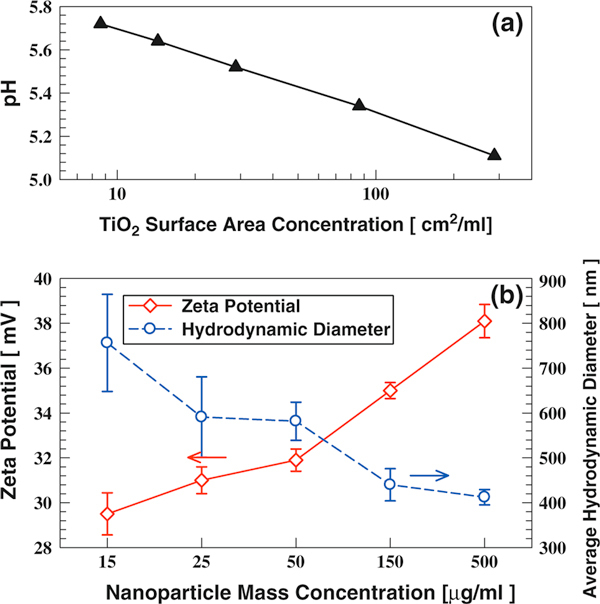
**The influence of nanoparticle surface area (mass concentration) on TiO_2_ (P25) dispersion characteristics: a pH; b zeta potential and hydrodynamic diameter**. Solvent is DI water.

Titania nanoparticle surface area in the dispersion affects both solution pH and dispersion properties. TiO_2_ (P25) nanoparticles with mass concentrations of 15, 25, 50, 150, and 500 μg/ml were dispersed in DI water. As the size of the particles in the sample is the same, the particle surface area is proportional to the particle mass concentration. As shown in Figure 3a, solution pH decreased as the particle surface area increased. When TiO_2_ nanoparticle is dispersed in water, its surface is covered by the hydroxyl group and extra hydrogen ions are produced (Eq. 1). Consequently, the solution pH decreases as more hydrogen ions are generated due to the increase in titania particle surface area. When particle mass concentration was increased from 15 to 500 μg/ml, the pH of the solution decreased from 5.7 to 5.1. Solution pH also became farther shifted from the TiO_2_ (P25) isoelectric point (6.2). Therefore, the dispersion zeta potential increased from 29 to 38 mV (Figure 3b). Though higher mass concentration often leads to larger hydrodynamic diameters, the average hydrodynamic diameter decreased from 756 to 412 nm, because the associated increase in the zeta potential (increase in repulsive force) prevented agglomeration. If the particle concentration is increased further, an increase in the average hydrodynamic diameter is expected, since the frequency of particle collision is a strong function of particle number concentration [54,55].

**Figure 4:**
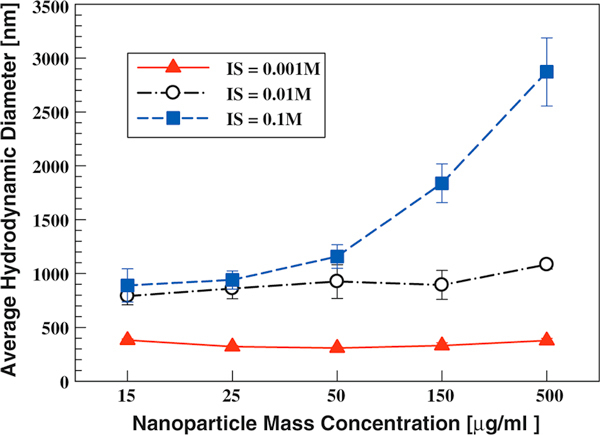
**TiO_2_ (P25) dispersion hydrodynamic diameter as a function of particle mass concentration at constant solution pH of 4 and different solution ionic strengths (0.001–0.1 M)**.

Particle concentration effect was further examined by fixing the solution pH at ~4. Three ionic strengths and five different mass concentrations were tested. As shown in Figure 4, the dispersion hydrodynamic diameter did not decrease with increasing particle surface area once the solution pH was fixed. At low solution IS, the dispersion hydrodynamic size remained similar with increasing particle concentration, because the electrostatic repulsive force helped to prevent agglomeration. At high solution IS, the increased particle number concentration led to enhanced coagulation rates and larger hydrodynamic diameters. At solution IS of 0.1 M and particle mass concentration of 500 μg/ml, the average hydrodynamic diameter was ~2,900 nm (large agglomerates).

### Primary Particle Size Effect

**Figure 5:**
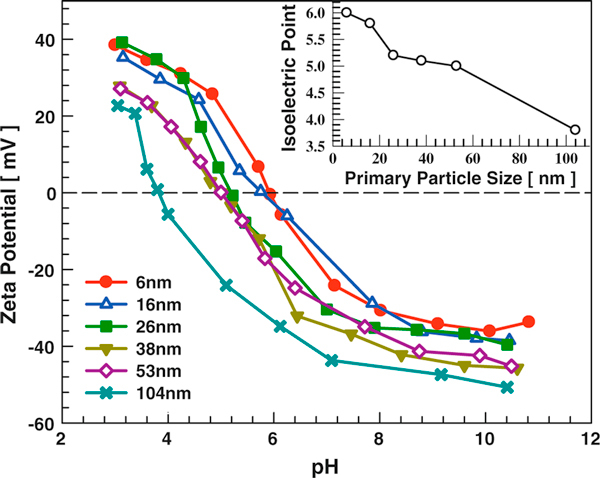
**The influence of anatase TiO_2_ primary particle size on dispersion zeta potential**. Solution IS is 0.001 M. Inset shows the titania nanoparticle dispersion isoelectric point (IEP) as a function of primary particle size.

The influence of primary particle size on the titania dispersion isoelectric point was tested using laboratory synthesized TiO_2_ nanoparticles. Anatase TiO_2_ samples of different sizes (6, 16, 26, 38, 53, and 104 nm) were tested using solutions with an IS of 0.001 M. As shown in Figure 5, the IEP of anatase TiO_2_ was found to be a function of primary particle size. When primary particle size increased from 6 to 104 nm, the IEP decreased from 6.0 to 3.8. It has been reported that different isoelectric points can be obtained for the same material depending on the synthesis method and experimental procedure [45,48,49]. This might explain why 27-nm TiO_2_ (P25) has an IEP of 6.2 while laboratory synthesized 26-nm TiO_2_ has an IEP of 5.2 (their crystal phases are also different, which will be addressed later). However, these six samples were prepared using the same synthesis technique, and the experimental procedures were the same. In addition, there was evidence suggesting that hematite nanoparticle IEP might vary with particle size [33], though only three sizes were examined in that study.

The size effect on dispersion isoelectric point might originate from size-related properties of nanoparticles. Several other activities of titania nanoparticles had been found to be size dependent. The photocatalytic activity of TiO_2_ nanoparticle was reported to be a function of particle size when the same total particle surface area was used [19,56]. Both in vitro and in vivo toxicities of anatase TiO_2_ (after normalized by surface area) were reported to be a function of particle size [16,32]. The adsorption affinity of metal (e.g., lead and cadmium) by TiO_2_ appeared to be size dependent [57,58]. As nanoparticle size decreases, the percentage of surface atom/molecule increases significantly. Particle electronic structure, surface defect density, and surface sorption sites also vary [7,59]. Consequently, both nanoparticle IEP and surface reactivity can become dependent on particle size. For instance, it has been observed that variations in the nanoparticle surface coordination environment lead to changes in the surface acidity constants [60,61].

**Figure 6:**
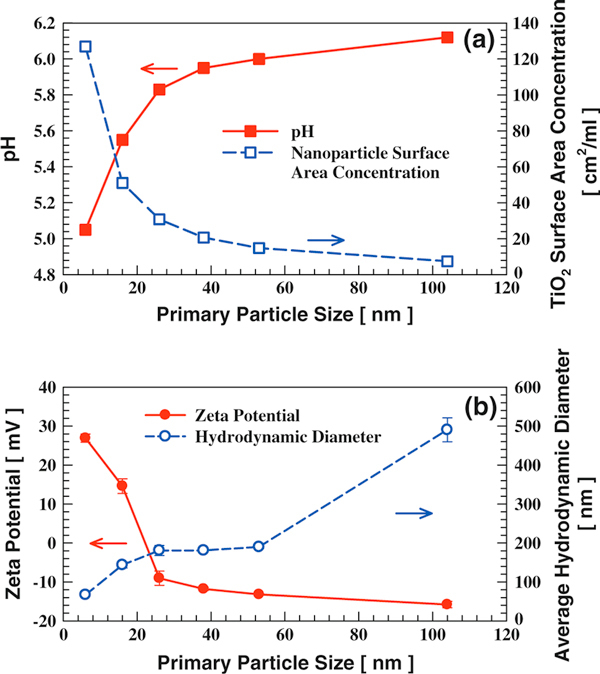
**Different sized anatase TiO_2_ nanoparticles dispersed in DI water with the same mass concentration of 50 μg/ml: a pH and surface area concentration; b dispersion zeta potential and hydrodynamic diameter**.

The effect of primary particle size on dispersion properties was examined by dispersing different sized anatase TiO_2_ in DI water. Since the same mass concentration (50 μg/ml) was used for all samples with differing particle sizes, the TiO_2_ particle surface area increased dramatically as particle size decreased (Figure 6a). Solution pH decreased with increasing particle surface area (as discussed earlier). Anatase TiO_2_ of 6 nm had the highest positive zeta potential due to its high IEP and low solution pH. A transition from positive to negative zeta potential happened between 16 and 26 nm. TiO_2_ of 104 nm has the highest negative zeta potential due to its low IEP and high solution pH. The average hydrodynamic diameter is not only a function of zeta potential and solution IS, but also a strong function of primary particle size. If no agglomeration occurs, i.e., the repulsive forces are completely dominant over the attractive forces, the hydrodynamic diameter should just reflect the primary particle size. The average hydrodynamic diameter increased from 67 to 490 nm as primary particle size increased from 6 to 104 nm (Figure 6b). The fact that the dispersion hydrodynamic diameter increment is not linearly proportional to primary particle size increment is due to particle–particle interaction that is affected by the dispersion zeta potential and IS. A detailed discussion of the reasons for the dispersion hydrodynamic diameter being larger than primary particle size can be found elsewhere [15].

### Nanoparticle Crystal Phase Effect

**Figure 7:**
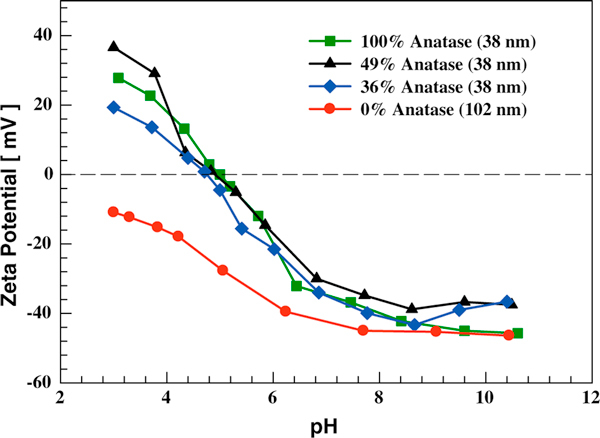
**The influence of TiO_2_ crystal phase on dispersion zeta potential**. Solution IS is 0.001 M.

The influence of titania crystal phase on dispersion isoelectric points was also examined using laboratory synthesized samples. Three 38-nm TiO_2_ samples with different anatase percentages (100, 49, and 36%; remainder being rutile) and one 102-nm rutile (100%) TiO_2_ sample were tested using solutions with IS of 0.001 M. For the three same sized TiO_2_ with different crystal structures, their dispersion isoelectric points (~4.8) were similar to each other (Figure 7). The IEP of 102-nm rutile TiO_2_ was lower than pH 3 such that the crossing point was not measured when pH range of 3–11 was used. The observation that the IEP of TiO_2_ at the same size is rather insensitive to the crystal structure is consistent with reports in the previous literature [49]. There are two possible factors accounting for the low IEP of the 102-nm rutile TiO_2_ sample. If the observed size-dependent IEP trend for anatase TiO_2_ is also valid for rutile TiO_2_, one would expect than 102-nm rutile has a lower IEP than that of 38-nm rutile. In addition, Figure 5 shows that 104-nm anatase TiO_2_ has an IEP of 3.8, while IEP of rutile with similar size (102 nm) is lower than pH 3. This might be related to the synthesis methods used—anatase TiO_2_ was directly synthesized using a flame aerosol reactor, while rutile TiO_2_ was prepared by annealing flame-synthesized 53-nm anatase TiO_2_ at 850°C using a furnace. Other studies [41,45] also found that metal oxides synthesized using different methods may have different isoelectric points.

## Conclusions

The effect of particle surface area, primary particle size, and crystal phase on TiO_2_ nanoparticle dispersion properties was tested. Solution pH and ionic strength play important roles in dispersion zeta potential and hydrodynamic size. Increasing titania particle surface area results in a decrease in solution pH. At fixed pH, an increase in titania mass concentration enhances the collision frequency between particles and leads to higher degree of agglomeration. In addition to synthesis method, TiO_2_ isoelectric point was found to be dependent on particle size. As anatase TiO_2_ primary particle size decreases, its IEP increases that also causes changes in dispersion zeta potential and hydrodynamic size. In contrast to particle size, it was demonstrated that TiO_2_ nanoparticle IEP is insensitive to crystal structure. These results have important implications both in developing nanomaterials for environmental applications and in performing nanotoxicological studies, because nanoparticle dispersion properties affect delivery and transport efficiency for both contamination remediation and for in vitro and in vivo toxicity tests.

## References

[B1] USEPA Science Policy Council 2007 USEPA, Washington, DC 20460

[B2] Lee MH Cho K Shah AP Biswas P Environ Sci Technol 2005 39 8481 10.1021/es0506713 16294891 16294891

[B3] Biswas P Wu CY J Air Waste Manag Assoc 1998 48 113 9517321 9517321 10.1080/10473289.1998.10463657

[B4] Tiwari V Jiang J Sethi V Biswas P Appl Catal A: Gen 2008 345 241 10.1016/j.apcata.2008.05.003

[B5] Biswas P Wu CY J Air Waste Manag Assoc 2005 55 708 16022411 16022411 10.1080/10473289.2005.10464656

[B6] Savage N Diallo MS J Nanopart Res 2005 7 331 10.1007/s11051-005-7523-5

[B7] Waychunas GA Kim CS Banfield JF J Nanopart Res 2005 7 409 10.1007/s11051-005-6931-x

[B8] Lee S Cho IS Lee JH Kim DH Kim DW Kim JY Shin H Lee JK Jung HS Park NG Kim K Ko MJ Hong KS Chem Mater 2010 22 1958 10.1021/cm902842k

[B9] Zhong P Que WX Nano-Micro Lett 2010 2 1

[B10] NSTC: Washington, D.C., 2008

[B11] Royal Society: London, 2004

[B12] Oberdorster G Maynard A Donaldson K Castranova V Fitzpatrick J Ausman K Carter J Karn B Kreyling W Lai D Olin S Monteiro-Riviere N Warheit D Yang H Part Fibre Toxicol 2005 2 8 10.1186/1743-8977-2-8 16209704 16209704 PMC1260029

[B13] Wiesner MR Lowry GV Alvarez P Dionysiou D Biswas P Environ Sci Technol 2006 40 4336 10.1021/es062726m 16903268 16903268

[B14] Oberdorster G Oberdorster E Oberdorster J Environ Health Perspect 2005 113 823 10.1289/ehp.7339 16002369 16002369 PMC1257642

[B15] Jiang J Oberdörster G Biswas P J Nanopart Res 2009 11 77 10.1007/s11051-008-9446-4

[B16] Jiang J Oberdörster G Elder A Gelein R Mercer P Biswas P Nanotoxicology 2008 2 33 10.1080/17435390701882478 20827377 PMC2935086

[B17] Carp O Huisman CL Reller A Prog Solid State Chem 2004 32 33 10.1016/j.progsolidstchem.2004.08.001

[B18] Chen X Mao SS Chem Rev 2007 107 2891 10.1021/cr0500535 17590053 17590053

[B19] Almquist CB Biswas P J Catal 2002 212 145 10.1006/jcat.2002.3783

[B20] Wei C Lin WY Zainal Z Williams NE Zhu K Kruzic AP Smith RL Rajeshwar K Environ Sci Technol 1994 28 934 10.1021/es00054a027 22191837

[B21] Warheit DB Webb TR Sayes CM Colvin VL Reed KL Toxicol Sci 2006 91 227 10.1093/toxsci/kfj140 16495353 16495353

[B22] Oberdorster G Phil Trans R Soc Lond A 2000 358 2719 10.1098/rsta.2000.0680

[B23] Powers KW Brown SC Krishna VB Wasdo SC Moudgil BM Roberts SM Toxicol Sci 2006 90 296 10.1093/toxsci/kfj099 16407094 16407094

[B24] Maynard AD Ann Occup Hyg 2002 46 197 10.1093/annhyg/mef063 12176765

[B25] Gilbert B Ono RK Ching KA Kim CS J Colloid Interf Sci 2009 339 285 10.1016/j.jcis.2009.07.058 19709669

[B26] Zeng H Singh A Basak S Ulrich KU Sahu M Biswas P Catalano JG Giammar DE Environ Sci Technol 2009 43 1373 10.1021/es802334e 19350906 19350906

[B27] Choi HS Liu W Misra P Tanaka E Zimmer JP Ipe BI Bawendi MG Frangioni JV Nat Biotechnol 2007 25 1165 10.1038/nbt1340 17891134 17891134 PMC2702539

[B28] Hoshino A Fujioka K Oku T Suga M Sasaki YF Ohta T Yasuhara M Suzuki K Yamamoto K Nano Lett 2004 4 2163 10.1021/nl048715d

[B29] Lockman PR Koziara JM Mumper RJ Allen DD J Drug Target 2004 12 635 10.1080/10611860400015936 15621689 15621689

[B30] Wu B Huang R Sahu M Feng X Biswas P Tang YJ Sci Total Environ 2010 408 1955 10.1016/j.scitotenv.2009.11.004 19931887

[B31] Jang HD Kim SK Kim SJ J Nanopart Res 2001 3 141 10.1023/A:1017948330363

[B32] Jiang J 2008 Washington University in St. Louis: St. Louis

[B33] Grassian VH J Phys Chem C 2008 112 18303

[B34] Braydich-Stolle LK Schaeublin NM Murdock RC Jiang J Biswas P Schlager JJ Hussain SM J Nanopart Res 2009 11 1361 10.1007/s11051-008-9523-8

[B35] He YT Wan JM Tokunaga T J Nanopart Res 2008 10 321 10.1007/s11051-007-9255-1

[B36] Jiang J Chen DR Biswas P Nanotechnology 2007 18 285603 10.1088/0957-4484/18/28/285603

[B37] Worathanakul P Jiang JK Biswas P Kongkachuichay P J Nanosci Nanotechnol 2008 8 6253 10.1166/jnn.2008.358 19205191 19205191

[B38] Dhumal SY Daulton TL Jiang J Khomami B Biswas P Appl Catal B: Environ 2009 86 145 10.1016/j.apcatb.2008.08.014

[B39] Thimsen E Biswas P AICHE J 2007 53 1727 10.1002/aic.11210

[B40] Delgado AV Gonzalez-Caballero F Hunter RJ Koopal LK Lyklema J J Colloid Interf Sci 2007 309 194 10.1016/j.jcis.2006.12.075 17368660

[B41] Kosmulski M Surface Charging and Points of Zero Charge 2009 CRC Press, Boca Raton

[B42] Stumm W Morgan JJ Aquatic Chemistry 1996 Wiley-Interscience, New York

[B43] Davis JA James RO Leckie JO J Colloid Interf Sci 1978 63 480 10.1016/S0021-9797(78)80009-5

[B44] Morrison ID Ross S Colloidal Dispersions: Suspensions, Emulsions, and Foams 2002 Wiley-Interscience, New York

[B45] Parks GA Chem Rev 1965 65 177 10.1021/cr60234a002

[B46] Derjaguin BV Landau LD Acta Physicochim URSS 1941 14 733

[B47] Verwey EJW Overbeek JTG Theory of the Stability of Lyophobic Colloids 1948 Elsevier, Amsterdam

[B48] Kosmulski M J Colloid Interf Sci 2009 337 439 10.1016/j.jcis.2009.04.072 19501839

[B49] Kosmulski M Adv Colloid Interf Sci 2002 99 255 10.1016/S0001-8686(02)00080-5 12509117

[B50] Grahame DC Chem Rev 1947 41 441 10.1021/cr60130a002 18895519 18895519

[B51] Widegren J Bergstrom L J Am Ceram Soc 2002 85 523 10.1111/j.1151-2916.2002.tb00127.x

[B52] Brant J Lecoanet H Wiesner MR J Nanopart Res 2005 7 545 10.1007/s11051-005-4884-8

[B53] Hunter RJ Zeta Potential in Colloid Science 1981 Academic press Inc, London

[B54] O'Melia CR Aquatic Chemistry 1995 American Chemical Society, Washington, DC 315 10.1021/ba-1995-0244.ch016

[B55] Friedlander SK Smoke, Dust, and Haze: Fundamentals of Aerosol Dynamics 2000 Oxford University Press, New York

[B56] Sclafani A Herrmann JM J Phys Chem 1996 100 13655 10.1021/jp9533584

[B57] Gao Y Wahi R Kan AT Falkner JC Colvin VL Tomson AB Langmuir 2004 20 9585 10.1021/la049334i 15491190 15491190

[B58] Giammar DE Maus CJ Xie LY Environ Eng Sci 2007 24 85 10.1089/ees.2007.24.85

[B59] Gilbert B Banfield JF Mol Geomicrobiol 2005 59 109

[B60] Bickmore BR Rosso KM Nagy KL Cygan RT Tadanier CJ Clays Clay Miner 2003 51 359 10.1346/CCMN.2003.0510401

[B61] Bullard JW Cima MJ Langmuir 2006 22 10264 10.1021/la061900h 17107031 17107031

